# Effect of Chemical Segregation and Surface Defect Formation on the Mechanism of the Aluminum Dendrite Growth

**DOI:** 10.1021/acsnano.5c20990

**Published:** 2026-04-01

**Authors:** Xiaodong Liu, Fatemehsadat Rahide, Tingting Yang, Penghan Lu, Helmut Ehrenberg, Sonia Dsoke, Rafal E Dunin-Borkowski, B. Layla Mehdi

**Affiliations:** 1 Department of Materials, Design & Manufacturing Engineering, 4591 University of Liverpool, Liverpool L69 3GQ, U.K.; 2 Institute for Applied Materials, 150232 Karlsruhe Institute of Technology, Hermann-von-Helmholtz-Platz 1, 76344 Eggenstein-Leopoldshafen, Germany; 3 Ernst Ruska-Centre for Microscopy and Spectroscopy with Electrons, Research Centre Juelich, Juelich 52425, Germany; 4 Department of Sustainable Systems Engineering, Albert-Ludwigs-University of Freiburg, Em-my-Noether-Straße 2, Freiburg 79110, Germany; 5 Fraunhofer Institute for Solar Energy Systems, Heidenhofstr. 2, Freiburg 79110, Germany; 6 Albert Crewe Centre, 4591 University of Liverpool, Liverpool L69 3GQ, U.K.

**Keywords:** scanning transmission electron microscopy, rechargeable aluminum battery, Al dendrite growth, chemical segregation, defect structure

## Abstract

Rechargeable aluminum batteries (RABs) are one of the most promising beyond lithium-ion battery chemistries. However, nonuniform dendrite growth during the cycling process remains an obstacle for practical application. In this work, we investigated different stages of the Al dendrite growth mechanism in an Al rechargeable battery system. The first stage of Al dendrite growth is a tip growing stage, where the chemical segregation behavior occurs at the center of the dendrite, with further strain concentration identified inside the chemically inhomogeneous regions; these chemical and strain inhomogeneities are attributed to the metal-corrosive electrolyte interaction. Furthermore, in the large dendrite growth stage, chemical segregation is not pronounced, while surface defect structures such as coherent and incoherent twin boundaries start to appear; these boundaries are connected through multiple stacking faults and migrate along the dendrite growing surface, which is believed to be one of the growth mechanisms for the large dendrite surface. This investigation provides an in-depth analysis of the microstructure evolution and changes occurring in Al dendrites during electrochemical disposition. This perspective creates opportunities for a more-tailed approach in designing future electrolytes and modifying anode surface to promote uniform ion deposition and lessen the safety concerns of Al dendrites in Al rechargeable batteries.

## Graphical abstract

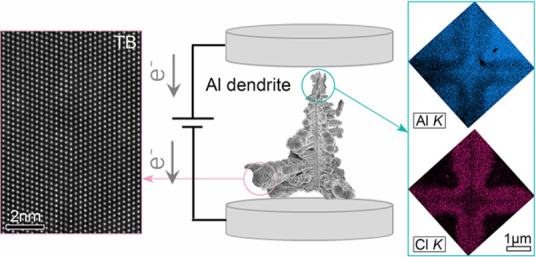

## Introduction

Rechargeable aluminum batteries (AlBs) are promising metal-based batteries, which exhibit superior energy density at a low cost,^1–3^ thereby having huge potential for energy storage applications. However, one of the main obstacles for practical application is the aluminum dendrite growth issues from corrosive electrolyte during the electrochemical plating and stripping processes.^4,5^ During the repeated cycling process, the nonuniform electrodeposition of aluminum results in the growth of sharp metallic dendrites, which can break the separator and cause cell short circuits, thereby leading to the failure of the cell. Therefore, it is of great importance to develop effective approaches to understand the aluminum dendrite growth process.

Various strategies of aluminum anode optimization were proposed to inhibit the growth of metallic dendrites or to further achieve the dendrite-free behavior in rechargeable AlBs.^6–9^ Effective methods are based on controlling the grain size and orientation of the aluminum anode,^10,11^ such as applying surface modifications (generating protective interface layers such as aluminophilic and oxide layers)^12–15^ and introducing a porous structure.^16^ Another promising way is through optimizing the electrolytes and thereby controlling the reaction between the metallic anode or dendrite and the electrolytes. Successful approaches such as incorporating electrolyte additives^7,17^ or optimizing the cation sites of the electrolyte^18–21^ have been proven to be helpful in inhibiting the aluminum dendrite growth. However, only few studies^22^ present the relationship between the anion sites (particularly for Cl-based anion sites from the electrolyte) and the anode/dendrite. Furthermore, current studies investigate the dendrite inhibition mechanisms through the perspective of electrochemistry, while only limited reports provide more straightforward evidence from crystallographic or microscopy perspectives to understand the dendrite growth mechanism. Therefore, it is imperative to gain an in-depth understanding of the microstructural evolution of aluminum dendrite during the electrochemical deposition.

In this work, we selected the most widely used [EMIMCl]:AlCl_3_ (ratio 1:1.5) ionic liquid (IL) electrolyte^4,9,23^ and performed the electrodeposition of aluminum, thereby growing the dendrites onto commercially available aluminum foil. Through combining the transmission Kikuchi diffraction (TKD) and aberration-corrected scanning transmission electron microscopy (STEM) techniques, we provide direct atomic-level evidence of electrolyte-related chemical segregation behavior inside the aluminum dendrite at different growing stages and further propose the conceivable growth mechanism of Al dendrites.

## Results and Discussion

Chronoamperometry (CA) was performed to electroplate Al on Al foil from the [EMIMCl]/AlCl_3_ electrolyte (as shown in Figure S1), and the recorded cathodic curve is presented in Figure S1. The oscillations were observed in reductive currents during CA, which is attributed to the reductive decomposition of the anions in the electrolyte.^9,24^ This can be further supported by the XPS spectra of Cl 2p transitions in our previous study,^4,9^ indicating the continued bonding of chlorine to both Al and [EMIM]. The current density is decreasing with increasing time; the lower current reduction rate after 2 h is due to the Al deposition covering the reaction surface and thereby reducing the active surface area of the Al substrate. Freshly deposited Al may also serve as a nucleation site, facilitating further deposition.^25^

The deposited Al dendrites exhibit different morphologies, such as spherical or cauliflower-like-shaped and Christmas tree-shaped dendrites (Figure S2). These variations in dendrite morphologies can be attributed to the combination of factors, including the magnitude and duration of applied voltage. Additionally, the characteristics of the aluminum foil (surface roughness, purity, and grain orientations) will also affect the dendrite morphologies.

Despite these diverse morphologies, we selected one of the most typical Christmas trees, typed dendrites, to investigate the crystallographic and chemical information during the electrodeposition process. Figure 1 shows the SEM image of a typical Christmas tree-type Al dendrite, with a length of approximately 300 μm. FIB lamellas were lifted out from the two directions, from the transverse plane (Figure 1a) and longitudinal plane (Figure 1b), which are vertical and parallel to the growth direction of the primary dendrite.

**Figure 1:**
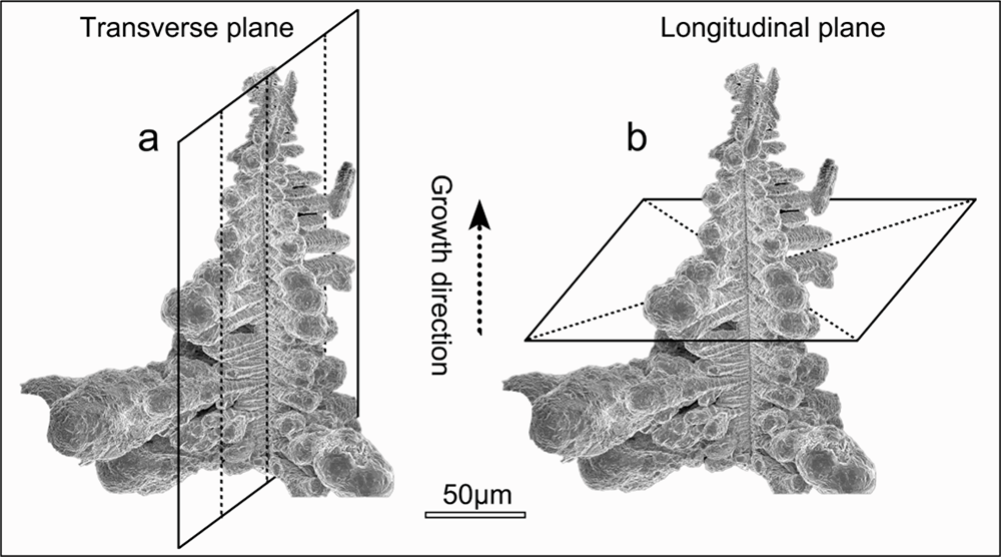
SEM image of selected typical Christmas tree-type Al dendrite and the FIB fabrication routes: (a) along the transverse plane and (b) along the longitudinal plane.

We first investigated the Al dendrite tip along the transverse plane (Figure S3). Figure S4 shows the cross-section view of the dendrite tip, where the spiky primary dendrite (with length approximately 16 μm and diameter less than 2 μm) is located at the center and the spindle-like secondary dendrites neatly growing on the side. STEM was employed to further understand the structure and the underlying growth mechanism of the dendrite tips. Figure 2a (BF STEM image) shows the center of the primary dendrite (marked by the white dash line) and part of the secondary dendrites (highlighted using the green arrows), where they possess vertical growth directions (primary dendrite growing from left to right, while the secondary growing from bottom to top). The TKD band contrast map (Figure 2d) strengthened the contrast of the dendrites, but it should be noted that these minor distortions (mainly from the origin of the secondary dendrites) are due to the relatively longer collection time for the TKD mapping. The TKD inverse pole figure (IPF) map (Figure 2e) exhibits a predominant single-crystal structure for the primary and secondary dendrites, and only a minor number of nano grains are formed inside the single crystal. In combination with the atomically resolved HAADF STEM image and corresponding FFT pattern along the <110>_Al_ zone axis from the single-crystal region (Figure 2b,c), the crystallographic information on the dendrite growth can be revealed: The primary dendrite is growing along the <001>_Al_ direction, while the secondary dendrites are growing toward the <1̅10>_Al_ direction, which are in good agreement with the reported typical Al dendrite growth behavior.^26^ Additionally, the TKD KAM map (Figure 2f) shows high-level misorientations at the primary dendrite center and the origins of the secondary dendrite, indicating the relatively high strain level at these regions.^27,28^ STEM EDX was employed along the <110>_Al_ zone axis to further investigate the origins (Figure 2g). The BF STEM image shows the center of the primary dendrite (region with higher misorientations); the corresponding EDX maps and quantitative EDX line profile (Figure S5) indicate that the distorted region is slightly depleted in Al but enriched with Cl, while the single-crystal region with few misorientations does not contain Cl. This can be further proved by atomically resolved EDX maps from both regions (Figure 2h,i), where the disorientated region contains not only Cl but also C and N compared to the regions with few misorientations (Figure 2j). Cl, C, and N are believed to originate from the [EMIMCl]:AlCl_3_ (chemical formula of C_6_H_11_ClN_2_:AlCl_3_) electrolyte. It is noted that despite these extra elements found inside the disorientated region, the atomic structure remains the same (face center cubic Al). However, partial irregular arrangement of atoms can be observed in the atomically resolved STEM image (at disorientated region) along the <110>_Al_ zone axis (Figure 2k); the corresponding geometric phase analysis (GPA) strain maps (Figure 2l–n) indicates the higher strain concentration compared to the pure Al region. This is in good agreement with the TKD KAM maps (Figure 2f), revealing that the Cl, C, and N impurities can cause severe strain concentration at the tip of the dendrite during the growth stage.

**Figure 2:**
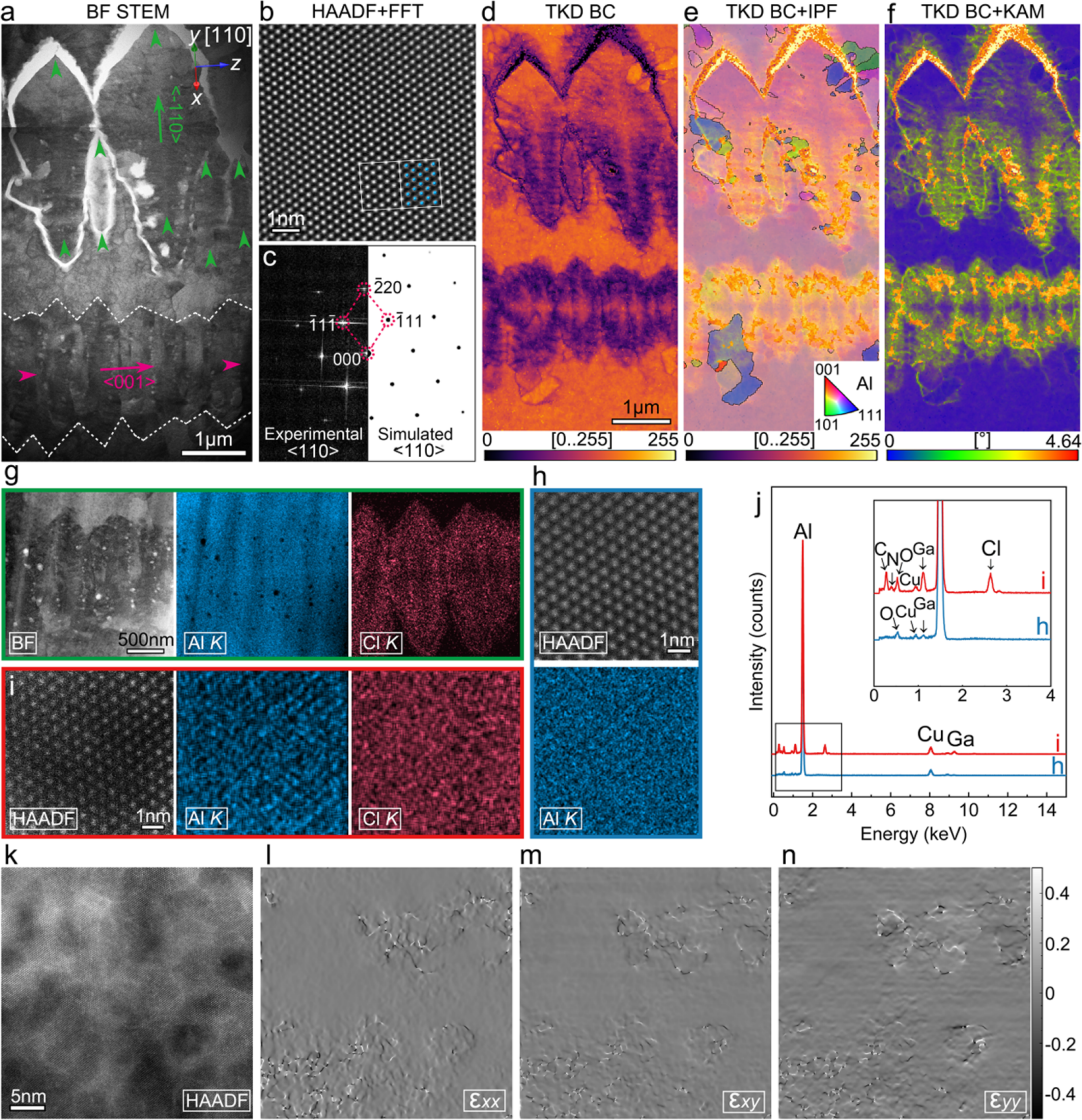
STEM analysis of the Al dendrite tip along the transverse plane. (a) BF STEM image of the cross-section view. (b) Atomically resolved HAADF STEM image of the Al dendrite along <110> zone axis. (c) Corresponding experimental FFT and simulated diffraction patterns. (d) TKD band contrast (BC) map. (e) TKD BC + inverse pole figure (IPF) map. (f) TKD BC + Kernel average misorientation (KAM) map. (g) STEM EDX maps of the center of the primary Al dendrite. (h, i) STEM EDX maps of the region (h) and region (i) from (g). (j) EDX spectra of the region I and j. (k) Atomically resolved HAADF STEM image of region (i). (l–n) Corresponding geometric phase analysis of the region in (k).

Following the investigation along the transverse plane, we also conducted a similar STEM analysis along the longitudinal plane, where we lifted out the lamella along the cross-sectional plane (indexed by the yellow arrow in Figure S6). Figure 3a shows the BF STEM image of the collected lamella; here, we can observe the center of the primary dendrite (bright and located at the center of the BF STEM image), and there are four branches growing along the perpendicular directions. On the two sides of the branches, a high-density hyperbranched seaweed structure can be observed, exhibiting different contrast in both BF STEM and TKD BC images (Figure 3c). The TKD IPF image (Figure 3d) indicates that the region is also predominantly single crystal with only one smaller grain growing between the top and left dendrite branches. It can be noted that the IPF map shows minor orientation differences along the seaweed structure, which can be further confirmed by the KAM map (Figure 3e), revealing the much higher lattice misorientation levels along the seaweed structure. The corresponding STEM EDX maps (Figure 3f) and quantitative line profile (Figure S7) show that the seaweed structure is enriched in Cl but depleted in Al, in good agreement with the chemically distorted region observed in the transverse plane (Figure 2g). Further comparison between the pure Al and the Cl-enriched area is presented in Figure 3g,h, indicating that the seaweed structure exhibits the same lattice structure with Al and contains not only Cl but also C and N (Figure 3i), also matching well with the transverse plane (Figure 2j). Additionally, minor segregation can be observed for Cl at the atomic level (Figure 3h), which might explain the contrast difference in the Cl-enriched area, as shown in Figure 3h,j. In Figure 3k, partial irregular arrangement of atoms in the Cl-enriched area is also distinguished, and the corresponding GPA maps (Figure 3l,m) confirm the higher strain level from the atomic scale, which correspond well with the TKD KAM results (Figure 3e and Figure S6).

**Figure 3:**
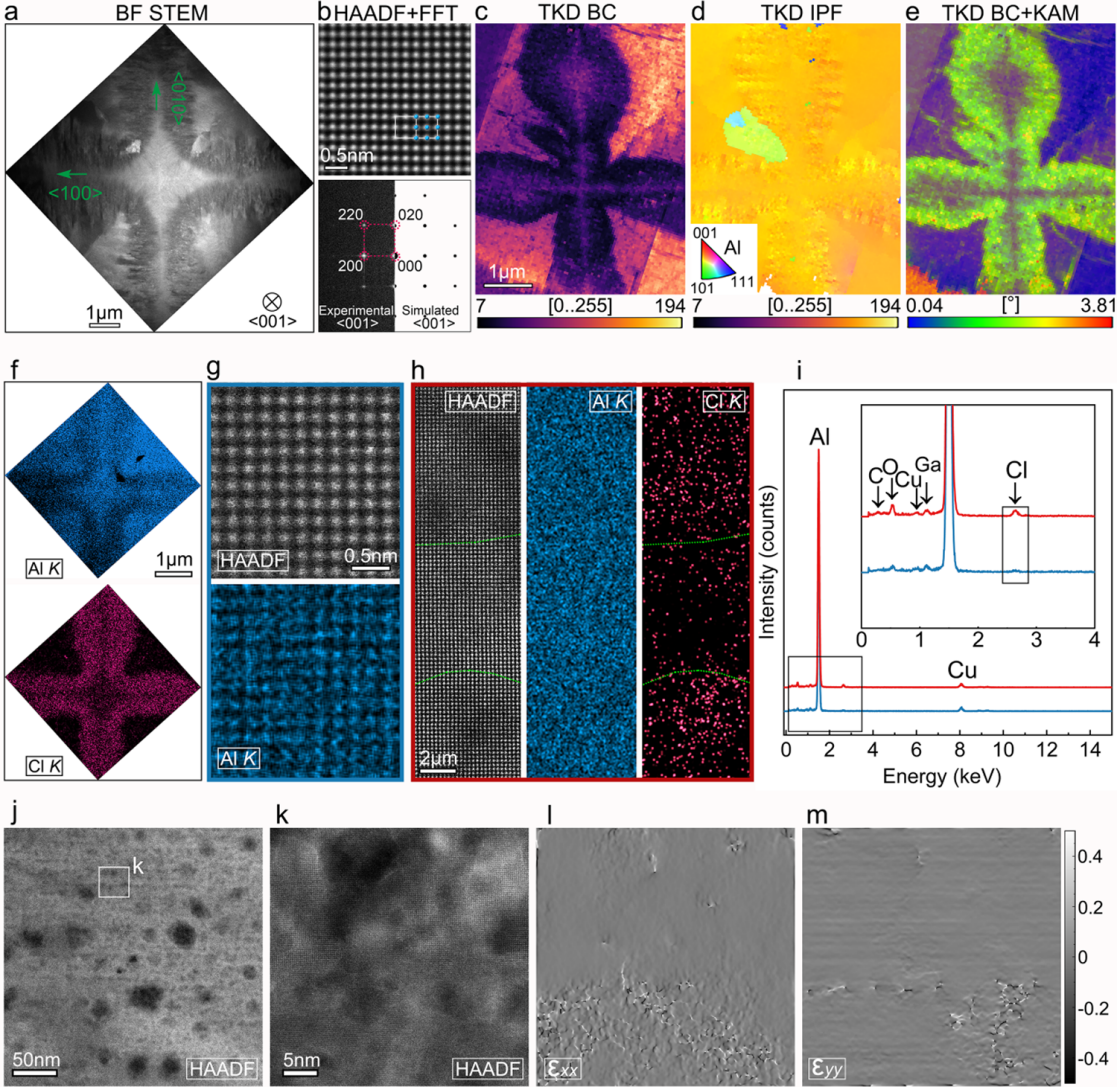
STEM analysis of the Al dendrite tip along the longitudinal plane. (a) BF STEM image of the cross-section view. (b, c) Atomically resolved HAADF STEM image of the Al dendrite along the <001> zone axis and the corresponding experimental and simulated diffraction patterns. (c) TKD band contrast (BC) map. (d) TKD inverse pole figure (IPF) map. (e) TKD BC + kernel average misorientation (KAM) map. (f) STEM EDX maps of the center of the primary Al dendrite. (g, h) STEM EDX maps of region (g) and region (h) from (f). (i) EDX spectra of region (g) and (h). (j, k) HAADF STEM images of the region (h). (l, m) Corresponding geometric phase analysis of the region in (k).

From the investigations along the transverse plane and longitudinal plane for the dendrite tips, severe element segregation behavior was observed, which is not common in most of the metal dendrite deposition processes. We ascribe the severe segregation to the interaction between the electrode and the electrolyte, as the electrolyte is the only source that can provide all of the segregated elements. The segregated C and N species correspond to the adsorbed ionic fragments generated by the electrochemical decomposition of EMIM^+^ cations. Literatures^29–31^ have shown that under specific electrochemical conditions (high cathodic overpotential or high current density), EMIM^+^ ions can decompose and produce C- and N-containing organic fragments. Although electrodeposition in this work was performed under constant voltage conditions, the electric field concentration and rapid tip growth are able to induce locally enhanced cathodic overpotential and current density, thereby promoting the electrochemical decomposition of EMIM^+^ ions and generating C- and N-containing organic fragments. In contrast, the segregated Cl can be attributed to the adsorbed or defect-associated chloride species. Although the chloride segregation in electrodeposited Al has not been directly quantified in the literature, it is well established that halide anions (including Cl^–^) from ionic liquids tend to accumulate and adsorb at electrified metal interfaces under strong cathodic polarization.^32,33^ Cl^–^ can also adsorb on metal surfaces with electronic interaction between the anion and surface atoms,^34^ indicating the weak nonstoichiometric coordination rather than lattice incorporation or new phase formation.

The segregated elements are located at the interface between the primary dendrite core and the interdendritic region, and this element distribution behavior is highly dependent on the local growth velocity of the aluminum interface. This is analogous to the continuous growth model in rapid solidification theory.^35^ In the growth stage, dendrite tips can reflect the initial growing stage of the dendrite and have a relatively smaller curvature radius, which are able to create a high overpotential and concentrated current density, thereby resulting in the rapid Al growth. When the local growth velocity exceeds the critical diffuse velocity, impurities are trapped at the interface between the primary dendrite core and the interdendritic region. Our results suggest that severe chemical segregation at the dendrite tip can also cause lattice misorientation and strain concentration, thereby leading to localized chemical and stress inhomogeneities. These inhomogeneities can destabilize the dendrite growth front, promoting the tip splitting and secondary branching as the system evolves toward minimizing the total Gibbs free energy,^36^ which can explain the well-developed secondary and multiple dendrite branches.

Apart from the interdendrite tip structure, the surface of the large Al dendrite arm (Figure S8) can also reveal the later growth stage of the dendrite, which was further investigated by the STEM techniques. Figure 4a shows a sequence of BF STEM images of the Al dendrite surface, where the white region (on the top) is the carbon and Pt deposition layers (as our samples were prepared by FIB techniques; these two protection layers were deposited to protect the surface of the dendrite). From Figure 4a, linear boundaries (highlighted by the white dashed line) can be observed along the dendrite surface; these horizontal growing boundaries show parallel relationships and show clear migration along the dendrite surface. The selected electron diffraction (SAED) patterns indicate that, compared to the inner dendrite (Figure 4c), the horizontal linear boundary shows another set of the diffraction patterns (Figure 4b); both sets of patterns are mirror symmetry to the {1–11}_Al_ planes, revealing that these horizontal linear boundaries are actually {111}⟨110⟩_Al_ twin boundaries (TBs). The structure of the TBs can be further confirmed by the atomically resolved HAADF and BF STEM images (Figure 4d); compared to the inner Al dendrite (bottom twin domain) (Figure 4e), the top domain and the bottom domain are mirror symmetry to the {1–11}_Al_ TB, indicating that these horizontal linear boundaries are {111}⟨110⟩_Al_ coherent TBs (CTBs).

**Figure 4:**
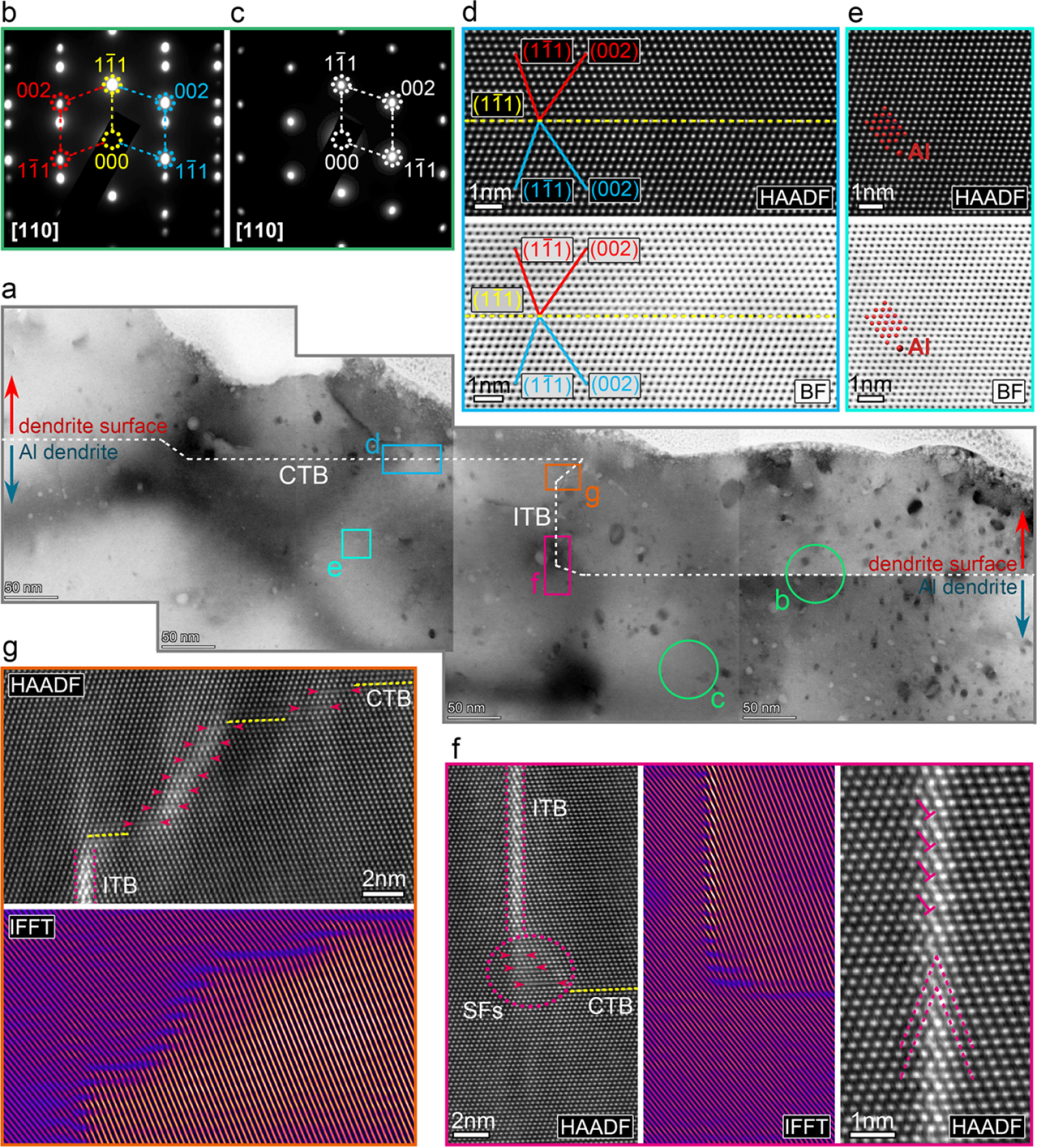
STEM analysis of the defect structure along the large dendrite arm surface: (a) BF STEM images of the Al dendrite surface. (b) (c) Selected area electron diffraction patterns of the region denoted by the green circles in (a). (d) HAADF and BF STEM images of the coherent twin boundaries denoted by a blue rectangular box in (a). (e) HAADF and BF STEM images of the Al dendrite along the [110] zone axis. (f, g) HAADF STEM images and corresponding IFFT images of the incoherent twin boundaries and the connection region between the ITB and CTB.

Apart from these horizontal linear boundaries, the vertical boundaries and other boundaries that connect the horizontal CTBs are also pronounced, where these boundaries are believed to support migration of the CTBs. The atomic structure of these boundaries is shown in Figure 4f,g. The vertical boundary (Figure 4f) separates the two twin domains, while the twin interface is not coherent; therefore, it is actually the incoherent twin boundary (ITB), in good agreement with the reported typical ITB in Al-based alloys.^37–40^ However, it is noted that the ITB presents brighter contrast compared to the matrix; this is attributed to the FIB fabrication-induced Ga impurities (Figure S9), as these Ga ions are tending to segregate to the boundaries.^41^ Additionally, Figure 4f,g also shows the connection routes between the ITB and CTB, and the HAADF STEM images and the corresponding IFFT images reveal that the junctions are actually composed of multiple stacking faults. Similarly, the junction between two parallel CTBs is also formed by multiple SFs (Figure S10). Furthermore, isolated SF density (Figures S11 and S12) exhibits clear variations between the CTB region and the junction region, where the isolated SF density sharply decreased near the junction region. As the junctions are structurally composed of ordered multiple SFs, this might indicate that isolated SFs generated near the junction regions are continuously absorbed and reorganized into the junction regions. This is consistent with the partial dislocation-mediated TB migration mechanism,^42,43^ in which sequential nucleation and glide of Shockley partial dislocations generate stacking faults that are incorporated into the boundary, thereby driving the CTB migration. This also matches well with the literature,^44^ supporting the plausibility of TB-mediated growth under electrochemical conditions.

## Conclusions

In this work, we focused on in-depth analysis of the Christmas-tree-type Al metal dendrites prepared by electrodeposition from the [EMIMCl]/AlCl_3_ electrolyte. We have performed detailed microstructural investigations of the two stages of the dendrite growth, including the early tip-growth stage and the later-stage growth of large dendrite arms. The chemical segregation behavior was observed at the initial growing stage, while surface defect structures were identified at the later growing stage.

During the initial growing stage, the dendrite tip is predominantly a single crystal. Severe chemical segregation was observed at the interface between the primary dendrite core and the interdendritic region, where these regions are enriched in Cl, N, and C elements and slightly depleted with Al. The unique distribution of these impurities can be attributed to the rapid growth velocity of the aluminum interface induced by the high overpotential and concentrated current density. However, the phase structure of the dendrite region remains the same, revealing that these segregated elements might correspond to adsorbed ionic fragments. Furthermore, the chemical segregation also induced a strain concentration at this region, thereby also increasing the surface energy of the dendrite tip and finally promoting the development of secondary and multiple dendrites. For the later growing stage, parallel linear defects such as CTBs and ITBs were found at the surface of the large dendrite arm; they are connected through a high density of stacking faults and thereby migrate along the dendrite surface, which is considered one of the growth mechanisms of the Al dendrite arm.

This work provides insights into microstructural evolution and chemical information on electrodeposited Al dendrite, which could guide the development of a dendrite-based optimization strategy for Al-based electrolytes to optimize electrodeposition, increasing the safety of Al dendrites in Al rechargeable battery systems.

## Materials and Methods

### Materials and Electrochemical Setup

In this work, the ionic liquid (IL) electrolyte was prepared by mixing 1-ethyl-3-methylimidazolium chloride ([EMIMCl]) (95%, Sigma-Aldrich) and AlCl_3_ (99.99%, Sigma-Aldrich) (with a molar ratio of 1:1.5 [EMIMCl]:AlCl_3_) inside an argon-filled (Ar-filled) glovebox (MBraun, O_2_ and H_2_O level <0.1 ppm). The resulting mixture formed a yellowish, clear liquid.

The electrochemical experiment was conducted inside an airtight and sealed TSC surface cell (TSC SC) (rhd Instruments GmbH & Co), which consists of a gold-plated thermo-block with an integrated Pt100 temperature sensor and a PEEK housing. Al foil (thickness of 0.075 and 0.025 mm, purity of 99.0%, Goodfellow) was taken as the working electrode (WE) for Al electrodeposition (with a geometric area of 0.28 cm^2^) (Figure S13); the glassy carbon (GC) disc (with a radius of 6 mm) served as the counter electrode (CE), and Al wire was used as a quasi-reference electrode (RE). The cell was assembled and disassembled inside an Ar-filled glovebox. Before each electrochemical experiment, the cell components were cleaned with ethanol and deionized water through an ultrasonic bath followed by a rinse in acetone. The components were then transferred to a glovebox for overnight vacuum drying at 323 K.

Similar to our previous investigations,^4,9^ chronoamperometry (CA) measurements were performed using a BioLogic potentiostat at a room temperature in a TSC surface cell. A constant voltage of −1 V was applied vs Al quasi-reference electrode to promote the electrodeposition of Al dendrite on the Al electrode in [EMImCl]/AlCl_3_ ILE. To remove residual electrolyte, the Al electrode was rinsed in anhydrous DMC inside an Ar-filled glovebox and subsequently vacuum-dried for 4 h at room temperature.

### Characterization

The morphology of the electrodeposited Al dendrite was observed using a Thermo Fisher Helios 5CX FEG scanning electron microscope (SEM), equipped with an Oxford Instrument SDD energy-dispersive X-ray (EDX) detector. The orientation of the dendrite was determined by off-axis transmission Kikuchi diffraction (TKD) techniques, using an Oxford Instrument Symmetry S3 Electron Backscatter Diffraction (EBSD) detector.

The nanoscale features of the electrodeposited Al dendrite were investigated by transmission electron microscopy (TEM). The bright-field (BF), dark-field (DF), and high-resolution TEM (HRTEM) images and selected area electron diffraction (SAED) patterns were collected using a JEOL JEM-2100Plus TEM (LaB_6_), operated at 200 kV. The scanning transmission electron microscopy (STEM) images of the dendrite morphology were obtained using a JEOL 2100F Cs-corrected dedicated STEM, operated at 200 kV. The atomically resolved STEM observations and EDX mappings were carried out using a Thermo Fisher Spectra 300 Cs-corrected STEM and a JEOL JEM-ARM300F2 GRAND ARM2 Cs-corrected STEM, operated at 200 kV. For Spectra 300 STEM, the BF and HAADF STEM images were collected simultaneously at the convergence angle of 21 mrad. For GRAND ARM2 STEM, the BF, LAADF, and HAADF STEM images were collected simultaneously at the convergence angle of 25 mrad.

Samples for TEM/STEM measurement were prepared by focused ion beam (FIB) techniques, employing a Thermo Fisher Helios 5CX dual-beam FIB SEM. The FIB fabrication parameters were optimized to reduce FIB-induced contamination (Figure S14). It should be noted that as the aluminum surface is air sensitive,^45^ the FIB-prepared TEM samples were transferred using a vacuum transfer holder and kept in a glovebox before loading to the TEM column.

## Supplementary Material



## Notes

Cathodic curve of the electrodeposition; SEM images of deposited dendrites; selected regions from the dendrite for FIB lift-out; TKD band contrast, IPF, and phase maps of the Al dendrite; STEM images and quantitative EDX line profiles of dendrite tip regions; STEM images and EDX maps for ITB structure; TEM and STEM images of the stacking faults; TEM and EDX maps of the pristine Al foil; and EDX spectrum of Al dendrite sample (PDF)
