# Supplementary material for: Effect of Chemical Segregation and Surface Defect Formation on the Mechanism of the Aluminum Dendrite Growth
Source: ACS Nano. 2026 Apr 1;20(14):11088–95. doi: 10.1021/acsnano.5c20990 (PMC13089874; doi:10.1021/acsnano.5c20990)
Supplement: Supplementary file 1 [file nn5c20990_si_001.pdf]

Supporting Information

Effect of Chemical Segregation and Surface Defects Formation on the Mechanism of the Aluminium Dendrite Growth

Xiaodong Liu[1†], Fatemehsadat Rahide[2†], Tingting Yang[3], Penghan Lu[3], Helmut Ehrenberg[2], Sonia Dsoke[2,4,5], Rafal E Dunin-Borkowski[3], B. Layla Mehdi[1, 6*]

[1]Department of Materials, Design & Manufacturing Engineering, University of Liverpool, Liverpool L69 3GQ, UK.

[2]Institute for Applied Materials, Karlsruhe Institute of Technology, Hermann-von-Helmholtz-Platz 1, 76344 Eggenstein-Leopoldshafen, Germany.

[3]Ernst Ruska-Centre for Microscopy and Spectroscopy with Electrons, Research Centre Juelich, 52425 Juelich, Germany.

[4]Department of Sustainable Systems Engineering, Albert-Ludwigs-University of Freiburg, Emmy-Noether-Straße 2, 79110 Freiburg, Germany.

[5]Fraunhofer Institute for Solar Energy Systems, Department of Electrical Energy Storage, Heidenhofstr. 2, 79110, Freiburg, Germany.

[6]Albert Crewe Centre, University of Liverpool, Liverpool L69 3GQ, UK.

[†]These authors contributed equally.

* Corresponding author: B. Layla Mehdi; blmehdi@liverpool.ac.uk.

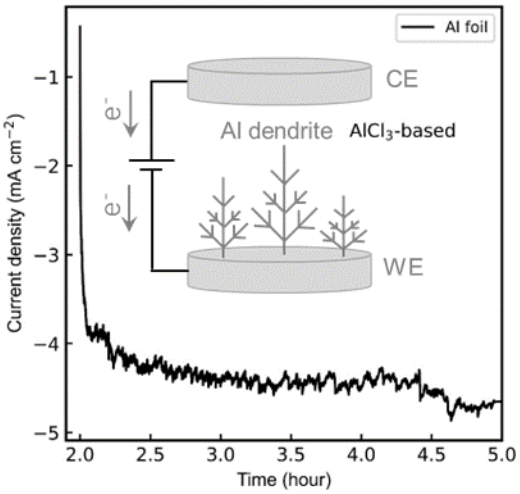

Figure S1. Recorded cathodic curve of the electrodeposition process.

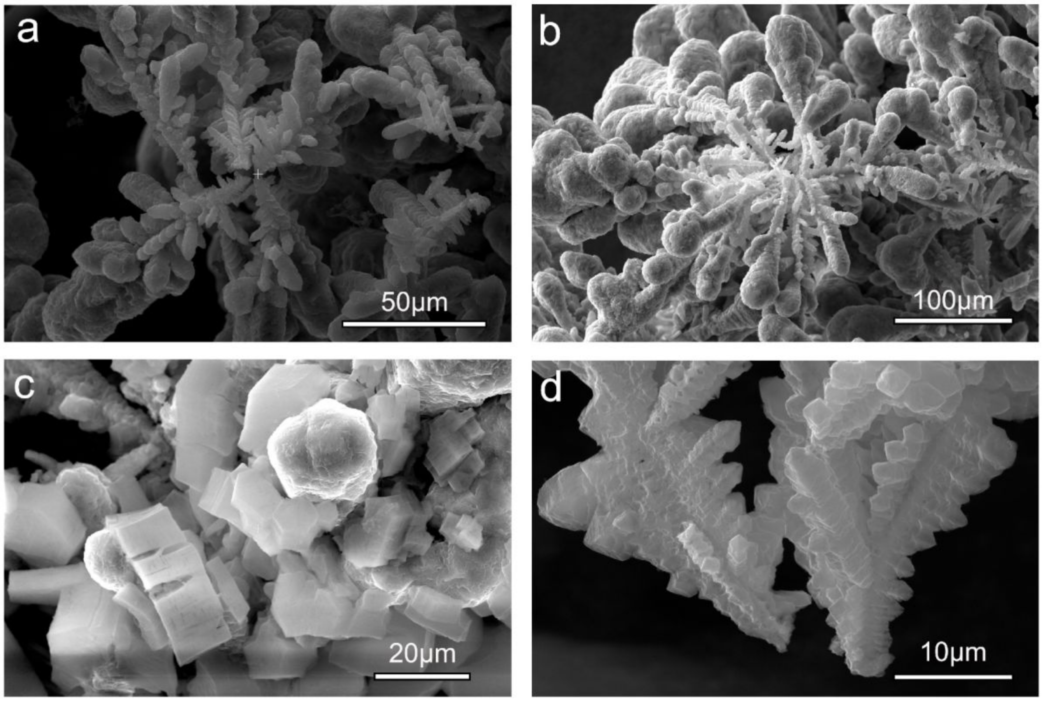

Figure S2. SEM images of deposited Al dendrites.

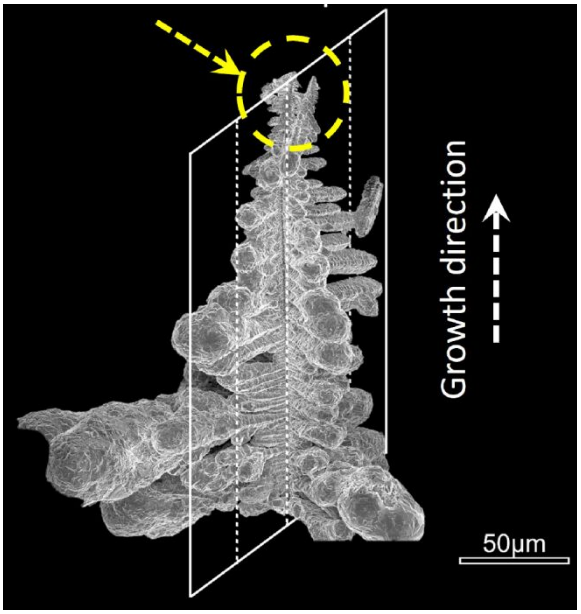

Figure S3. The selected region from the dendrite tip for FIB lift out.

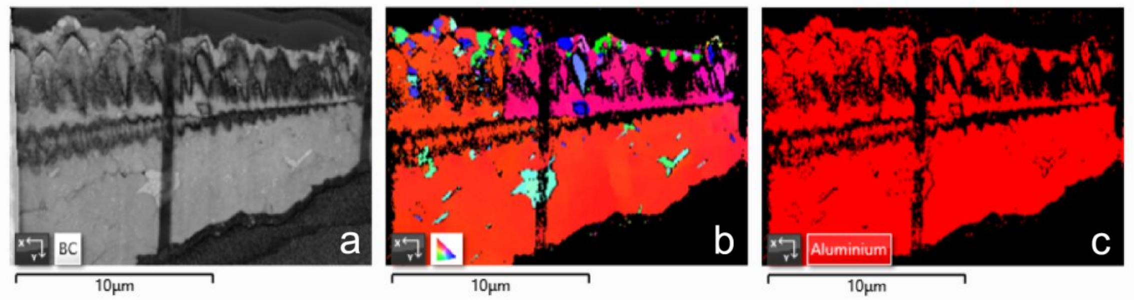

Figure S4. TKD band contrast, IPF and phase maps of the cross-section of as-prepared Al lamella.

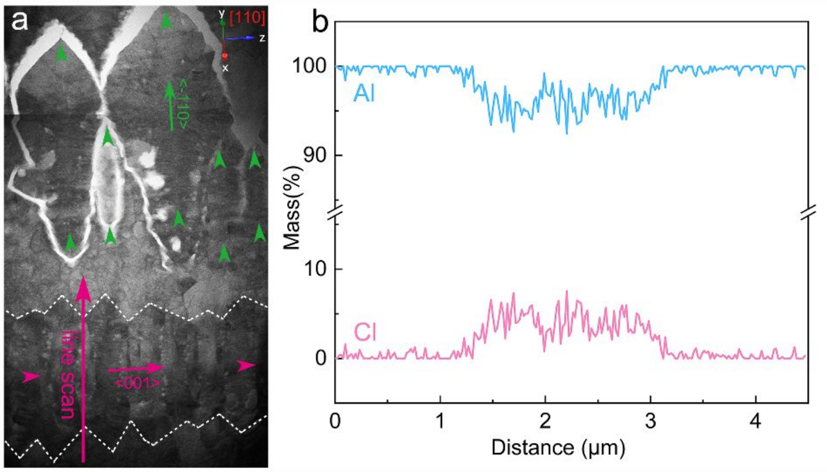

Figure S5. (a) BF STEM image of dendrite tip along the transverse plane; (b) quantitative EDX line profile from (a).

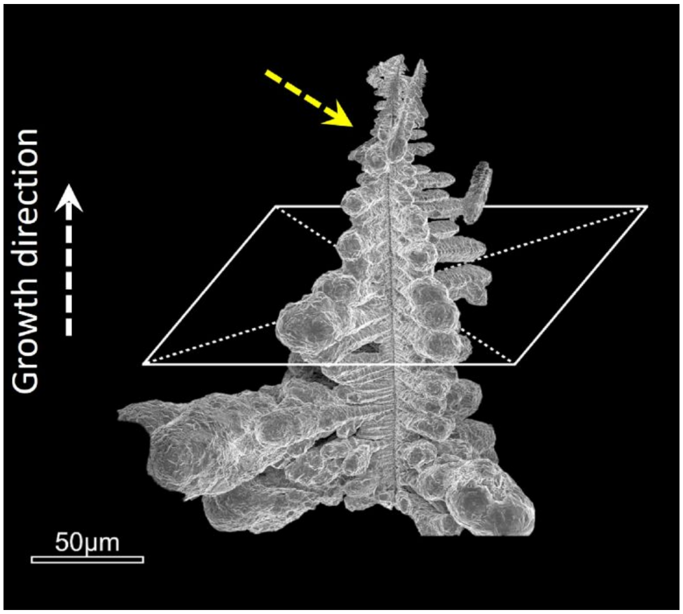

Figure S6. The selected region from the dendrite tip for FIB lift out.

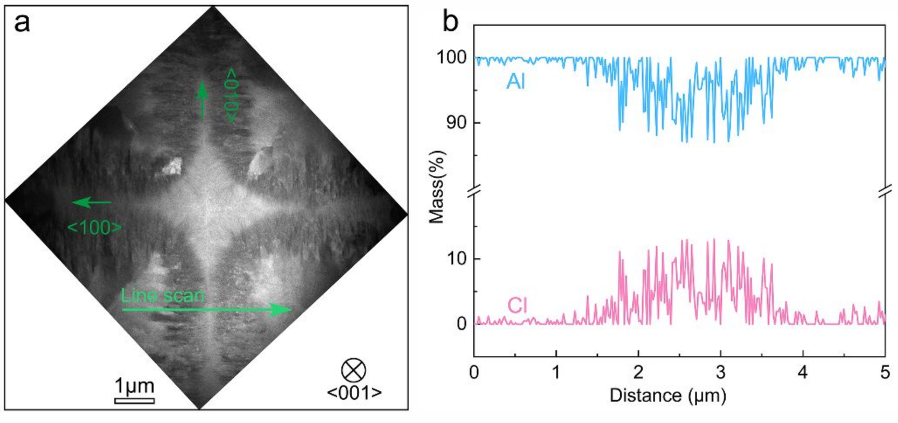

Figure S7. (a) BF STEM image of dendrite tip along the longitudinal plane; (b) quantitative EDX line profile from (a).

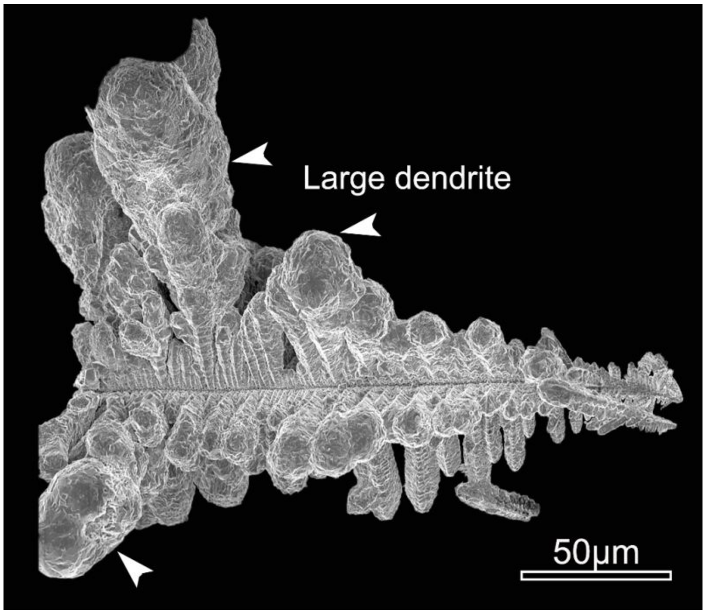

Figure S8. The selected region from the large dendrite arm for FIB lift out.

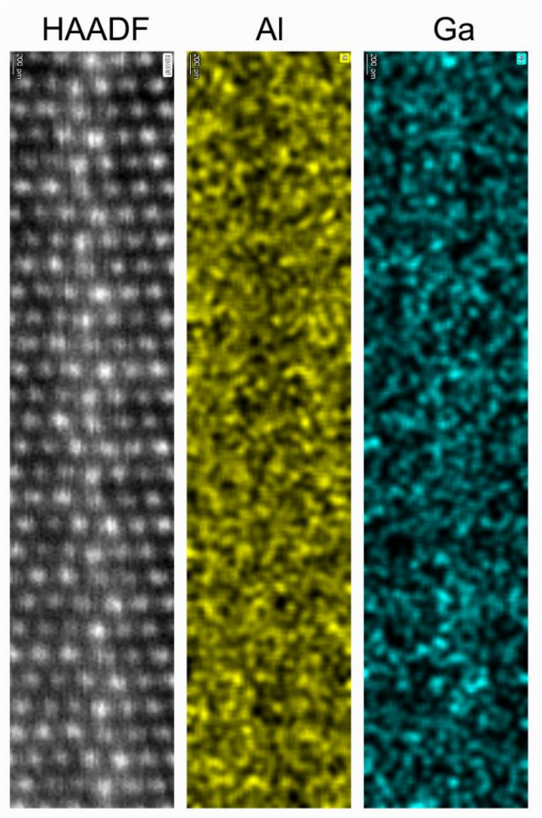

Figure S9. HAADF STEM image and corresponding EDX maps for the ITB structure.

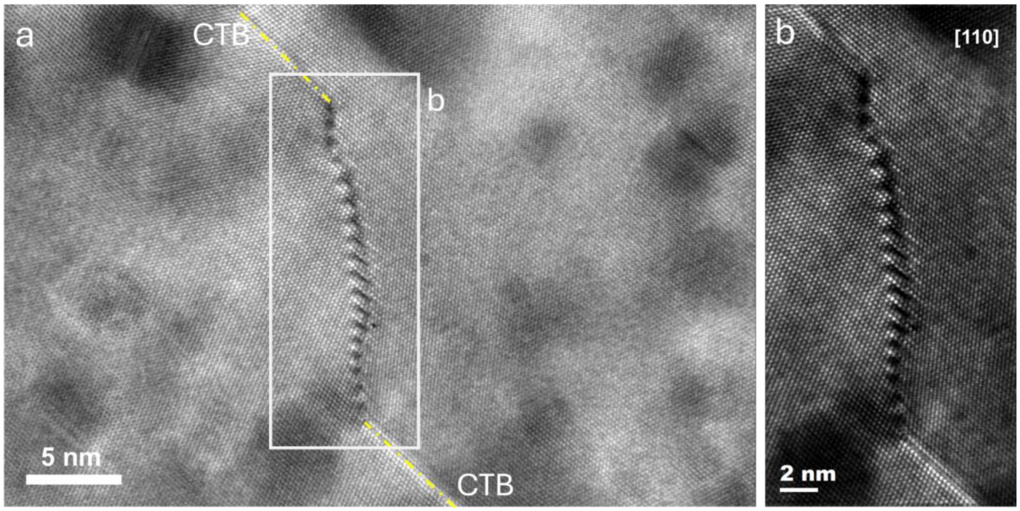

Figure S10. HRTEM image of the multi-SF structure.

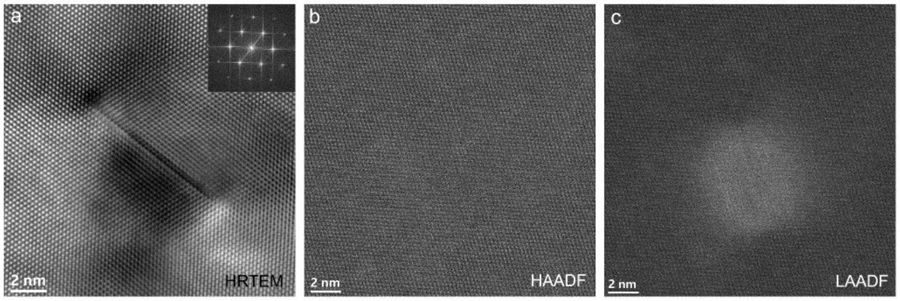

Figure S11. (a) HRTEM image of a stacking faults in the sample; (b)(c) HAADF and LAADF STEM images of stacking faults in the sample.

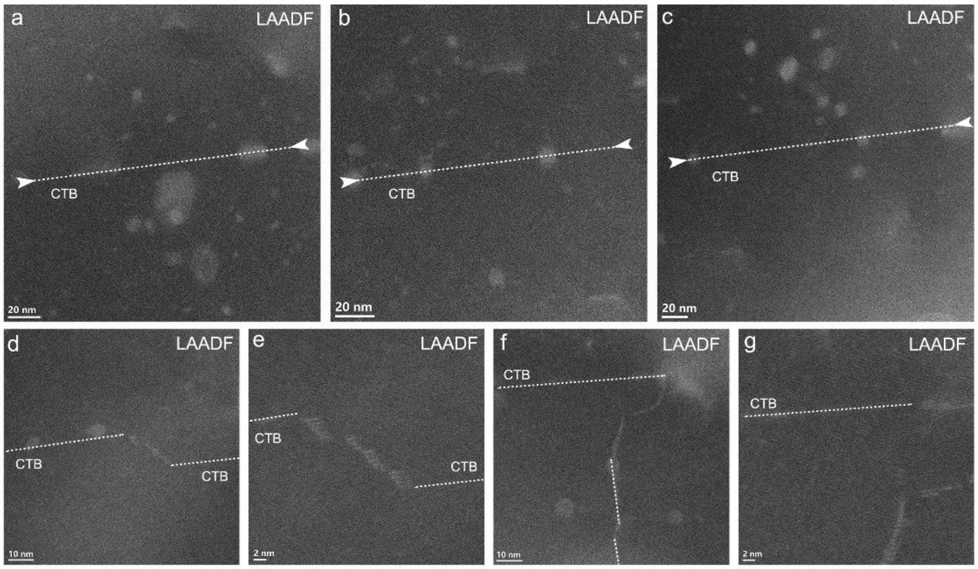

Figure S12. LAADF STEM images of stacking faults near the (a-c) CTB and (d-g) the migration junctions.

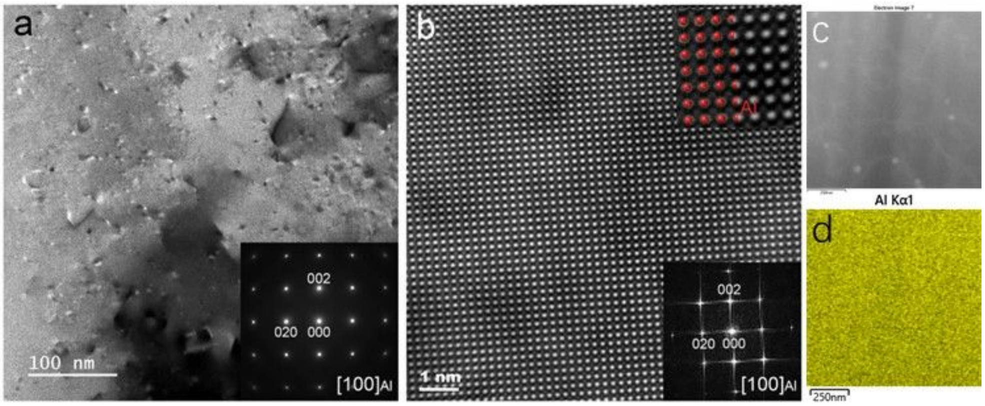

Figure S13. TEM analysis of the pristine Al foil along the [100] zone axis. (a) BF image and SAED pattern of the Al foil; (b) Noise-filtered HRTEM image and corresponding FFT pattern of the Al foil; (c) HAADF STEM image of the pristine Al foil; (d) STEM EDX mapping of Al element.

During the FIB preparation process, sample was subsequently rough milled at 30kV/0.79nA and 30kV/0.23nA; the following progressive thinning process was carried out at 16kV/0.15nA and 8kV/66pA; an ion beam of 5kV/15pA was applied during the final thinning process; after milling, the lamella was polished using 2kV/9pA ion beam to minimize the generation of amorphous layer. Through reducing both accelerating voltage and the ion beam current, the Ga implantation is expected to be effectively suppressed. Additionally, STEM EDX was conducted on the Al dendrite core, and the EDX spectrum was present in **Figure S14**, where the intensity ratio of Ga L/Al is approximately 0.011. This is significantly lower than the intensity ratio (0.024) from the sample prepared by conventional Ga FIB parameters (thinning at 30kV and polishing at 5kV) and the value is also comparable to the counts ratio after further nanomilling (0.016) [1], indicating that the Ga contamination is effectively suppressed in our sample.

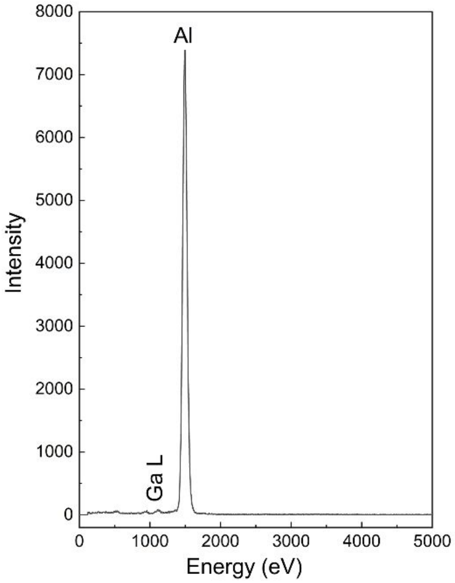

Figure S14. STEM EDX spectrum from the Al dendrite sample prepared using optimized FIB parameters.

[1] Unocic, A.; Mills, J.; Daehn, S. Effect of gallium focused ion beam milling on preparation of aluminium thin foils. Journal of microscopy. 2010, 240(3), 227-238.
